# Internet of Things (IoT)-Enabled Elderly Fall Verification, Exploiting Temporal Inference Models in Smart Homes

**DOI:** 10.3390/ijerph17020408

**Published:** 2020-01-08

**Authors:** Grigorios Kyriakopoulos, Stamatios Ntanos, Theodoros Anagnostopoulos, Nikolaos Tsotsolas, Ioannis Salmon, Klimis Ntalianis

**Affiliations:** 1 School of Electrical and Computer Engineering, Electric Power Division, Photometry Laboratory, National Technical University of Athens, 9 Heroon Polytechniou Street, 15780 Athens, Greece; 2 Department of Business Administration, University of West Attica, Thivon 250, Egaleo, 122 44 Athens, Greece; thanag@uniwa.gr (T.A.); ntsotsol@uniwa.gr (N.T.); isalmon@uniwa.gr (I.S.); kntal@uniwa.gr (K.N.); 3 Department of Infocommunication Technologies, ITMO University, Kronverksiy Prospekt, 49, St. Petersburg 197101, Russia

**Keywords:** elderly and impaired, healthcare, Internet of Things (IoT), fall verification, temporal inference model, smart homes

## Abstract

Everyday life of the elderly and impaired population living in smart homes is challenging because of possible accidents that may occur due to daily activities. In such activities, persons often lean over (to reach something) and, if they not cautious, are prone to falling. To identify fall incidents, which could stochastically cause serious injuries or even death, we propose specific temporal inference models; namely, CM-I and CM-II. These models can infer a fall incident based on classification methods by exploiting wearable Internet of Things (IoT) altimeter sensors adopted by seniors. We analyzed real and synthetic data of fall and lean over incidents to test the proposed models. The results are promising for incorporating such inference models to assist healthcare for fall verification of seniors in smart homes. Specifically, the CM-II model achieved a prediction accuracy of 0.98, which is the highest accuracy when compared to other models in the literature under the McNemar’s test criterion. These models could be incorporated in wearable IoT devices to provide early warning and prediction of fall incidents to clinical doctors.

## 1. Introduction

The elderly and impaired population will soon live in smart homes [1]. Such homes provide a pleasant and safe place for seniors. Individually, safety is considered a priority service, under the concept of smart healthcare [2]. However, daily emergency incidents will also continue to occur due to seniors’ human nature. Current Internet of Things (IoT) technology provides methods and models to prevent time-critical situations and emergency incidents [3]. Besides, such technology enables analytic models to infer whether an incident is an emergency or not. Clinical doctors will be able to utilize these models at a given emergency incident proactively and to provide immediate first aid to elderly and impaired persons. At this point, it is noteworthy that deep learning has paved the way for massive breakthroughs in the healthcare field. The discovery of groundbreaking architectures such as hierarchical computing architecture (HiCH), when blended with concepts like the convolutional neural network (CNN), enables IoT devices to step beyond the limitations of inaccuracy in a wireless body area network (WBAN). In parallel, machine learning algorithms such as C4.5, C5.0, KNN, and EM, promote the missing value analysis and the generation of decision trees, thereby making the working module/architecture much more efficient in its artificial intelligence (AI) upgrades [4].

This research focused on fall and lean over incidents of seniors in smart homes. While we treated a lean over as a daily, routine, voluntary movement, we differentiated a fall as an emergency incident, which needs further actions to be undertaken by seniors’ clinical doctors. IoT provides many components to input an analytical model capable of inferring and verifying fall incidents [5,6,7]. We propose the use of wearable altimeter sensors adopted by seniors to feed the models. Although in the literature, the use of IoT altimeter sensors is not a new approach, the impact of this research is the incorporation of temporal inference models for fall incident verification. Such models’ inputs can utilize altitude information of the individual, provided by the wearable sensor, such as the time required for falling to the floor, thereby inferring whether the incident is a lean over or a fall. 

Anonymous, real, and synthetic data were mined from Internet sources to deploy and verify the proposed temporal models. Specifically, real data from YouTube for fall and lean over incident videos were used, being processed to extract valuable input information to the proposed models. The results are promising for inferring and providing early warning of a fall incident. Clinical doctors can exploit such knowledge with access to the temporal models, offering prompt and accurate first aid services to seniors. 

IoT and edge computing have been recently considered as essential tools for visual object tracking at smart city applications. However, the adoption of IoT and edge computing necessitates demanding data collection, communication, and processing, along with high requirements for computing power and memory space, which severely prevent systematic and accurate tracking [8]. Real-Time Internet of Things (RT-IoT) is an evolutionary tool of the IoT, where a real-time communication over the Internet is achieved. In this respect, a global inter-networking of devices and physical things can be performed in real-time for the remotely controlling and automating of various jobs. However, in the case of missing the deadline of RT-IoT tasks, it may lead to hazardous situations, such as a human loss [9]. Therefore, designing and implementing a cloud-based novel architecture—aimed at IoT simulation and formal verification for a typical RT-IoT application—could develop a real centralized server in order to evaluate, to monitor, and to track real-time scheduled jobs and different IoT tasks within a smart space from anywhere [9].

From a safety and defense viewpoint of IoT, cyber-attacks, mainly data breaches and identity theft, are growing; thus, necessitating real-time connected devices to support sufficient security and defense in an integrated way [10]. In the relevant literature, an intelligent intrusion-detection system tailored to the IoT environment was developed: a deep-learning algorithm can detect malicious traffic in IoT networks through simulating and providing evidence of scalability and interoperability between various IoT-running protocols upon network communication [11].

An important application of IoT in the healthcare sector was introduced under the conceptualization of Internet of Medical Things (IoMT) [4]. IoMT envisages the interconnection of healthcare devices and sensors towards a broader spectrum of applications, relying on medical data processing and storage. Besides, machine learning and deep learning algorithms applied in the healthcare domain allow health professionals to monitor, diagnose, focus, and highlight the region of the problem and propose the required and accurate solution in the shortest duration possible [4].

Due to the reality that falls (especially in elderly persons) are a significant health problem worldwide, reliable fall detection systems can promptly mitigate the negative consequences. Consequently, contemporary research in the field of fall detection and verification of the elderly and impaired population is extensive. Among the critical challenges and issues reported in the literature is the difficulty of an accurate comparison between fall detection systems and machine learning techniques for detection [12]. 

In response to a few multimodal datasets developed upon different human activities, including publicly available falls, Martinez-Villasenor et al. presented the UP-Fall Detection Dataset. The authors decided to choose a 10-fold configuration based on relevant research and everyday practices reported in machine learning [12]. The dataset was adaptable enough to summarize full information from wearable sensors, ambient sensors, and vision devices. They stated that such a dataset could support human activity recognition and machine learning research communities to compare their fall detection solutions fairly. Furthermore, their dataset provides experimental possibilities for the signal recognition, vision, and machine learning community [12].

A prototype of a fall detection system by using accelerometer and gyroscope based on a smartphone was developed by Rakhman et al. [13]. This system can be used to provide an alert when a fall is verified. Furthermore, Tamura et al. [14], developed a wearable airbag that incorporates a fall detection system, which uses both acceleration and angular velocity signals to trigger inflation of the airbag. In another application, an adhesive sensor system worn on the skin automatically detected human falls based on a tri-axial accelerometer, a microcontroller, and a Bluetooth low energy transceiver [15]. Concerning recognition of falls from a silhouette, this can be achieved by incorporating video to segment the individuals from the background [16]. Fall verification is achieved by combining Microsoft Kinect and a two-stage fall detection system. Such a system is based on decision tree inference to detect the fall incident [17].

Human fall detection is inferred by a precise method based on indoor visual surveillance. Such a system incorporates the Gaussian mixture model (GMM) to exploit the foreground objects to perform pattern recognition [18]. Detection of falls by elderly persons is achieved by using a floor sensor, thereby enhancing electric near field imaging [19]. Activity fall detection is also achieved by using Doppler radar. Said method incorporates wavelet transformation to infer the fall incident [20]. 

Machine learning approaches can detect falls of elderly persons by exploiting real-world falls. Such methods infer a fall by incorporating lumbar sensors to capture the elderly population’s daily activity [21]. Wearable sensors can provide information to specific models to collect and analyze data streams and infer a fall detection incident [22]. A wearable biomedical signal measurement terminal is also able to perform automatic fall detection by feeding specific inference models [23]. Generally, wearable technologies can feed machine-learning models, such as feature selection and classification, to perform activity recognition and fall prediction [24]. Activity detection and early warning pre-impact fall detection can be achieved by using wearable devices to build certain dynamic threshold models [25]. IoT and Bluetooth capabilities can enable wearable low power models for efficient pre-fall detection and prediction [26]. Machine learning can be also used to evaluate fall characteristics, which can support a monitoring system for the strategic plan and fall prediction of the elderly and impaired population [27].

Fall detection of the elderly population can be inferred by incorporating barometric pressure and tri-axial accelerometer sensors of daily movement in smart homes [28]. Fall incident detection in real-time can be performed by a model which incorporates a wearable tri-axial accelerometer. Such a model can send emergency help messages to specific home care infrastructure in the case of a fall incident [29]. Moreover, tri-axial accelerometer sensor models can be used to predict fall detection by providing longitudinal fall-risk estimation [30]. A spectral analysis model of accelerometer sensor signals, which exploit certain directed-routine, can be used to calculate the fall-risk estimation for early warning of a fall incident [31]. The fall event detection model, which uses 2-D information, such as trunk angular velocity and trunk angle, can infer a prior-to-impact fall incidence of the senior population [32].

A portable device was incorporated to monitor the fall incidents of elderly persons, which integrates a microcontroller, a 3-DOF accelerometer sensor, a GSM/GPRS modem, and an embedded fall detection algorithm to exploit cascade posture recognition [33]. Power consumption is a constraint of wearable technology, which can be treated by selecting power-efficient signal features, thereby enabling a low-power fall detector [34]. A model can identify and inform back-end infrastructure for unconscious and conscious falls of elderly persons from heights above ground level [35]. Wi-Fi devices can be used to design and implement such a model, which can infer an indoor accurate fall incident in real-time with contactless and low-cost behavior [36]. A wearable altimeter sensor can feed an IoT-enabled model, which can verify if an incident is a fall or a lean over an elderly and impaired individual living in a smart home [37]. In a similar study, it was signified that fall prediction and prevention could be achieved by a model which evaluated the fall stimuli of the elderly in a split-belt treadmill. Such a model can exploit deferential velocities of the individuals [38]. Freefall datasets can also be used for fall prediction. Such datasets are fed into fall probability models, which incorporate supervised feature learning to infer a possible fall incident proactively [39].

Extensive research has also been performed in the area of fall prediction to detect a fall incident, [40] proactively. Specifically, fall prediction can be verified by analyzing fall risk assessments of the elderly, considering combinations of risk factors, and data produced by wearable sensors [41]. Body-worn inertial sensors can be used to explore the differences in semi-free-living gait between activity on stairs and on a regular, flat floor surface in regard to elderly and impaired daily activities [42]. A single standing time model was also used for predicting the fall risk of the elderly population [43].

Smartphone sensors are incorporated to assess certain fall risk factors, such as a decline in balance, reduced lower limb strength, and fear of falling. Such factors can predict possible fall incidents by sensors [44]. Similarly, a detection mechanism, which utilizes an accelerometer sensor in a smartphone, is used to measure the elderly movement and detect if a fall incident has occurred [45]. A method which uses a smartphone electronic compass and a tri-axial accelerometer is used to detect fall accidents. Such an approach incorporates positioning information and proposes a rescue system in case of a fall accident [46]. Inertial sensors can also be incorporated in certain models, which exploit near-fall scenarios to provide pre-impact fall detection [47].

A literature review in the area of lean over incidents considers areas of research relative to lean over detection and lean over prediction of an upcoming incident. Technologies used are wearable or implant sensors and devices, and embedded sensors and cameras located in smart home infrastructure. However, sparse research has been directed to the area of verification of an incident as a fall or a lean over movement. There is an architectural proposal for a healthcare system in a smart home, based on the architecture tradeoff analysis method (ATAM), wherein a timely diagnosis of environmental incidents and health risks can cause benefit the security, interoperability, and cost reduction of healthcare services [48]. At ATAM, scenarios are examined to meet the quality requirements. Specifically, whenever processed data of environmental sensors (gas leakage) or health sensors (falling elderly) show non-normal indications, a warning message is sent, and the elderly person’s family is notified. Whenever data are revealing an emergency case, then a prescription and a warning message will be sent to the data center to inform the elderly’s family and to call an ambulance. Subsequently, static data (such as personal information of elderly occupants) are constant and non-changeable, whereas dynamic data (such as medical profile of elderly occupants) are changeable [48].

Differentiating and verifying fall from lean over aims to understand the daily activity of an elderly and impaired individual. This study is a research extension of previous work [37], while incorporating temporal inference models for fall incident verification. Altitude and temporal information, provided by a wearable altimeter sensor, feed the proposed models to contribute to determining whether the incident is a lean over or a fall. 

The structure of the paper is as follows. The materials and the procedures incorporated in the proposed temporal inference modeling are described at Section 2. Then, the method derived by the experiments performed and the models’ outputs are addressed in Section 3. In Section 4, the results are summarized. Section 5 conducts a discussion on the results, and Section 6 concludes the paper with future work proposals.

## 2. Materials

Assume $t_{F}$ and $t_{B}$ as the fall and lean over times of an incident, respectively. Let h≥0 be the height in which the wearable device of the elder individual is located. It is defined that in case of h=0 the senior is considered to have reached the floor surface, either for a fall or a lean over incident. Assume also that $t_{F}$ and $t_{B}$ follow a normal probability distribution function (PDF), such as $N_{F}\;\left(\mu_{F},\sigma_{F}\right)$ and $N_{B}\;\left(\mu_{B},\sigma_{B}\right)$. We define the parameters of the PDFs experimentally to be $N_{F}\;\left(\mu_{F},\sigma_{F}\right)<N_{B}\;\left(\mu_{B},\sigma_{B}\right)$, since it is assumed that fall time of and individual is considered less than lean over time. This is explained because a fall incident conceptually is considered as a free fall, while a lean over incident is considered as a fall with an initial velocity and negative acceleration. We propose two classification models, namely, CM-I and CM-II, to evaluate if an incident is a fall or a lean over (bend), given certain experimentally defined normal PDFs of fall and lean over time.

### 2.1. Classification Model I (CM-I)

Given individual normal PDFs, i.e., $N\;\left(\mu_{F},\sigma_{F}\right)$ and $N\;\left(\mu_{B},\sigma_{B}\right)$, we consider the equation

(1) $$r=\frac{\mu_{F}+\mu_{B}}{2},$$

as the classification criterion of CM-I. Assuming an incident with time defined as $t_{I}$, if $t_{I}<r$, the incident is classified as a fall, or else it is classified as a lean over (bend). The conceptual model for CM-I is provided in Table 1. 

### 2.2. Classification Model II (CM-II)

Consider $t_{F}$ and $t_{B}$ as vectors of consecutive $t_{F}$ and $t_{B}$ time measurements, such as $t_{F}=\left\{t_{F}^{0},\ldots ,t_{F}^{j},\;\ldots ,t_{F}^{n}\right\}$, and $t_{B}=\left\{t_{B}^{0},\ldots ,t_{B}^{j},\;\ldots ,t_{B}^{n}\right\}$, where j is the j-th time measurement in which holds that h≠0 (i.e., the individual has not performed a fall or a lean over), while n is the n-th time measurement in which holds that h=0 (i.e., the individual has performed a fall or a lean over). Given an incident with $t_{I}=\left\{t_{I}^{0},\ldots ,t_{I}^{j},\;\ldots ,t_{I}^{n}\right\}$, the following equations are used:

(2) $$t_{fall}^{j}=\left|t_{I}^{j}-t_{F}^{j}\right|$$

and

(3) $$t_{bend}^{j}=\left|t_{I}^{j}-t_{B}^{j}\right|,$$

where $t_{fall}^{j}$ and $t_{bend}^{j}$ are the time classification criteria, of CM-II, used to classify the incident. Concretely, $t_{fall}^{j}$ and $t_{bend}^{j}$ are the time distances of a given incident $t_{I}^{j}$ with regard to $t_{F}^{j}$ and $t_{B}^{j}$ at a certain j-th time measurement, respectively. If $t_{fall}^{j}<t_{bend}^{j}$, then in the classification vector is considered to occur a fall incident at the j-th time measurement; i.e., fall←fall+1. It holds that the opposite in case of $t_{fall}^{j}>t_{bend}^{j}$ where the j-th time measurement is classified as a lean over incident; i.e., bend←bend+1. At the n-th time measurement, it is considered that h=0; i.e., if an elderly person’s wearable device is considered to have reached ground height, fall and bend quantities are compared. If fall>bend, then the incident is classified as a fall; else it is classified as a lean over. The conceptual model for CM-II is provided in Table 2.

### 2.3. Evaluation Metrics

Certain evaluation metrics are defined here, which were to assess the accuracy of the proposed classification models CM-I and CM-II. In case of CM-I, the classification accuracy is defined as follows:

(4) $$a=\frac{s_{p}+s_{n}}{s_{p}+q_{p}+s_{n}+q_{n}}$$

where $s_{p}$ and $s_{n}$ are the true positives and true negatives, and $q_{p}$ and $q_{n}$ are the false positives and false negatives for classified incidents of CM-I, respectively. Concretely, for classification model CM-II, it holds that the classification accuracy is defined as:

(5) $$a^{\prime }=\frac{s_{p}^{\prime }+s_{n}^{\prime }}{s_{p}^{\prime }+q_{p}^{\prime }+s_{n}^{\prime }+q_{n}^{\prime }}$$

where $s_{p}^{\prime }$ and $s_{n}^{\prime }$, are the true positives and true negatives, while $q_{p}^{\prime }$ and $q_{n}^{\prime }$ are the false positives and false negatives for classified instances of CM-II, respectively. 

Classification accuracies a, and $a^{\prime }$ of the proposed models take values in the interval [0, 1], where 0 indicates a poor accuracy, while 1 implies a high accuracy. A classification model is considered superior to another in the case that they are both applied and evaluated by the same datasets, and one reaches accuracy greater than the accuracy of the other. 

Consequently, a model is superior to another if false positives and false negatives are eliminated. However, since machine learning is an empirical science, the quality of the training dataset effects the quantity of prediction accuracy. An efficient model has less false positives and false negatives based on the creation of a more realistic normal PDF for the variable measured according to a certain training dataset. So, the better the normal PDF produced (i.e., trained by the model), the small the number of false positives and false negatives that will be observed, and a higher prediction accuracy will result.

## 3. Methods

We performed specific experiments to assess the accuracy of the proposed classification models. Specifically, we analyzed 86 videos randomly retrieved from YouTube: 41 depict a fall incident, while the remaining 45 describe lean over movements of elderly and impaired individuals living in smart homes. The experimental parameters are presented in Table 3. 

Videos were randomly captured form YouTube by using the embedded search engine with search keywords such as: (1) senior or elderly or impaired fall incident, and (2) senior or elderly or impaired lean over incident. We performed video analysis with Power Director video processing software. The NTSC standard was used to set up the video frames, where each video instance was decomposed to 30 frames per second (fps). Height (h) was measured in cm, and time (t) was measured in seconds. The information extracted from videos was used to feed the proposed classification models CM-I and CM-II. 

Evaluation of the models was performed by incorporating 10-fold cross-validation. We repeated the evaluation process for 100 iterations to minimize statistical error. We used 90 percent of the dataset for training the models, while the remaining 10 percent was used for validation. Specifically, we used 37 fall and 41 lean over incidents for training the models (i.e., 78 incidents), while the remaining incidents (4 fall and 4 lean over incidents) were used for testing the models of the original dataset.

**Figure 1:**
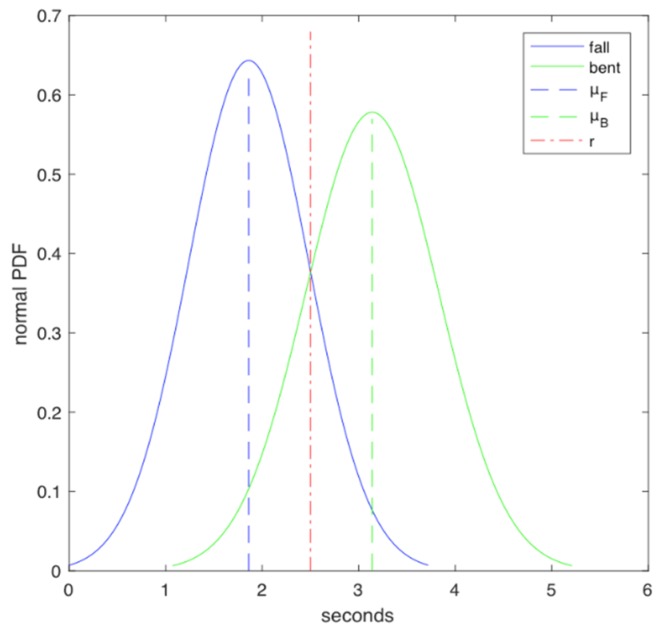
Normal probability distribution function (PDF) of $t_{F}$ (fall values) and $t_{B}$ (lean over values).

We found experimentally that height, h, is defined in the interval between [0, 1.72] cm, wherein 1.72 cm is the maximum height of an impaired senior, and 0 cm implies that the elder has reached the floor surface. Accordingly, fall time, $t_{F}$, is defined in the interval between [1.47 and  2.25] seconds; lean over time, $t_{B}$, takes values in the interval between [2.66, 3.62] s. It was found experimentally that normal PDF of $t_{F}$ is described by $N_{F}\;\left(1.86,\;0.39\right)$, where $\mu_{F}=1.86$, and $\sigma_{F}{}^{2}=0.39$ s. In addition, normal PDF of $t_{B}$ is described by $N_{B}\;\left(3.14,\;0.48\right)$, where $\mu_{B}=3.14$, and $\sigma_{B}{}^{2}=0.48$ s. According to Section 3, for the classification model CM-I it holds that $N_{F}\;\left(1.86,\;0.39\right)<N_{B}\;\left(3.14,\;0.48\right)$, while from Equation (1), it holds that the quantity $r=\frac{1.86+3.14}{2}=2.5$ s. See Figure 1 (red dotted line).

**Figure 2:**
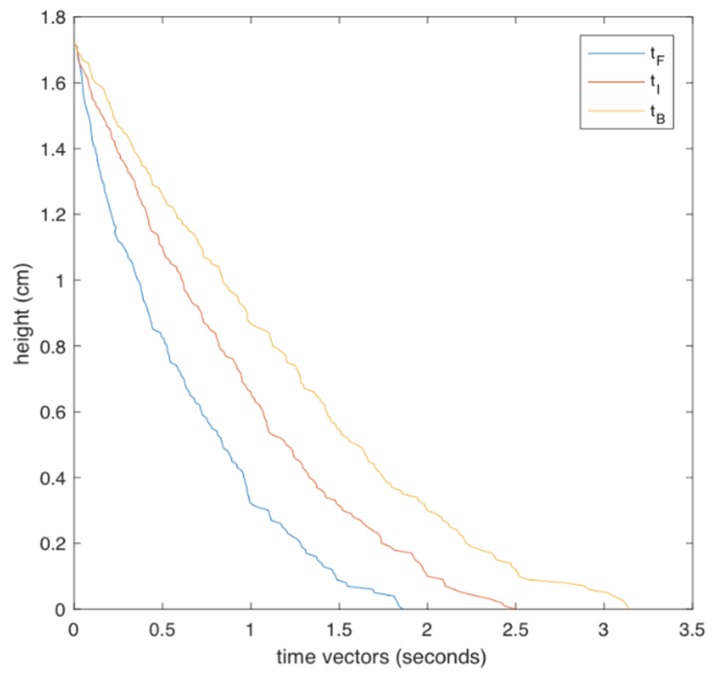
Time vectors of $t_{F}$ (fall values), $t_{I}$ (model values), and $t_{B}$ (lean over values).

Concretely, for the classification model CM-II it was found experimentally that $t_{I}$ follows the values described in Figure 2. 

Note that the final value of $t_{I}^{n}$, which was observed for h=0, is close to the quantity r of CM-I (2.5 s). This means that both models converge at the value r at the end of the incident. However, CM-II is more efficient, since it evaluates the whole history of the incident incrementally, not only at the end of the incident, as proposed by CM-I.

## 4. Results

**Figure 3:**
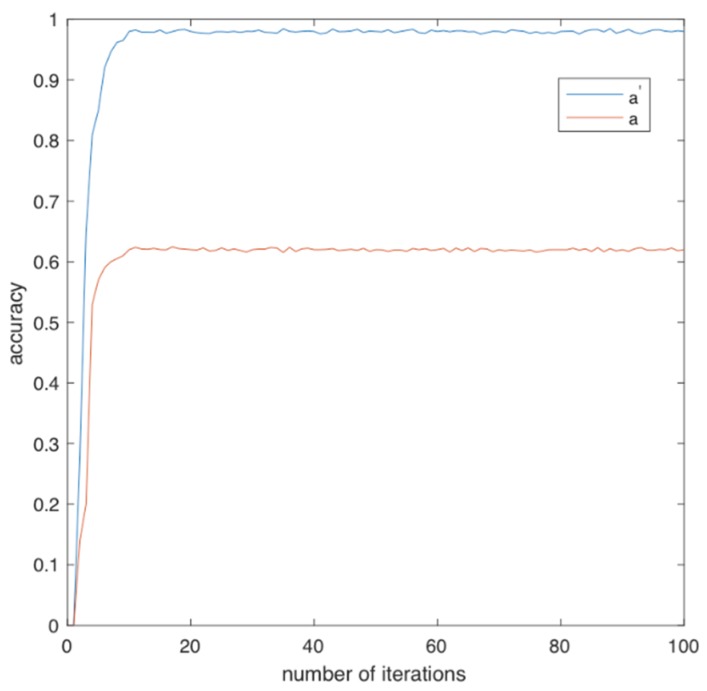
Accuracies a and $a^{\prime }$.

Execution of the experiment gave individual values for a and $a^{\prime }$ evaluation metrics. To minimize statistical error, we performed 100 iterations of the experiment. See Figure 3. 

Specifically, we can observe that the accuracy $a^{\prime }$ of the classification model CM-II is higher than the accuracy a of the classification model CM-I. $a^{\prime }$ reaches the value of 0.98, while a converges in value 0.62. This is explainable, since in CM-I, the research approach is statically based on the classification criterion r, which is calculated only once when normal PDF of $t_{F}$ and $t_{B}$ are defined experimentally. In this case classification model CM-I does not consider the incremental stochastic history of the incident as CM-II does. 

Concretely, in case of CM-II the model evaluates and computes classification accuracy $a^{\prime }$ of the instance incrementally, thereby tracking the senior’s movement behavior. CM-II compares each time measurement $t_{I}^{j}$ incrementally with the quantities $t_{F}^{j}$ (fall-time) and $t_{B}^{j}$ (lean over time), which is more efficient than CM-I. However, to prove the efficiency of CM-II over CM-I, we applied McNemar’s test to evaluate the statistical significance of the prediction accuracies achieved by both models [49]. According to McNemar’s test the prediction accuracies of the proposed models are statistically significant, and since the prediction accuracy of CM-II is significantly higher than that of CM-I, it holds that CM-II is better than CM-I. So, it is proven that CM-II is superior to CM-I; thus, it is proposed for adoption in case of elderly and impaired persons’ fall verification.

Furthermore, the proposed model CM-II was compared with other models in the literature to assess its efficiency. Specifically, we compared our model with the model in [13], which achieved accuracy 0.95. The model in [18] had accuracy 0.94, while the [20] approach reached accuracy 0.91. The proposed model in [18] has an accuracy 0.86, while the [25] model has an accuracy 0.96. Approach in [27] reached accuracy 0.97, while the [28] model achieved accuracy 0.89. Equivalently, we applied McNemar’s test to evaluate the statistical significance of the prediction accuracies achieved by all models. According to McNemar’s test, the prediction accuracies of the proposed models are statistically significant. Thus, the prediction accuracy 0.98 observed by our CM-II is statistically significantly higher compared with the prediction accuracies of all the other models. The comparison of the proposed model CM-II with other models in the literature is presented in Table 4. 

## 5. Discussion

From a research viewpoint, IoT-enabled elderly fall verification is not a simple and straightforward issue. Contrarily, it entails a robust theoretical background and the adoption of a suitable methodological approach. It is indicatively stressed that the IoT sensor data is generated from various heterogeneous devices, communication protocols, and data formats that are enormous in nature. Hence, learning approaches, data acquisition, semantic annotation, resources data extraction, semantic reasoning, and clustering can address the problem of sensor data integration and analysis in IoT healthcare data [50]. In a similar study it was argued that modelling features of object tracking on lightweight computing devices, involves limited computing capacity and memory space. Thus, there is a need for a fast algorithm fitted to such devices [8].

From a benchmark viewpoint, a random forest (RF) algorithm presented the best results in almost all experiments [12].

*Random forest (RF). This is an “ensemble method made of decision trees, in which an input is processed through the forest of decision trees and computes the output class as the mode of the response class given by the trees.” This technique is employed in many fall detection and activity recognition systems*.

(source: [12], page 16)

From a performance and operability viewpoint, it is noteworthy that deep learning models contain multiple processing layers proficient in learning significant features of data without the need for a domain level capability. On the other hand, traditional machine learning methods usually need a sizable amount of domain-level knowledge to perform classifications [4]. It is also critical for researchers to determine the specific features of machine learning task, since some related works are recognizing only the fall/not fall classifications while other works attempt to classify a more versatile spectrum of activities. Therefore, this diversified classification of data and metrics has yet to be verified with respect to relevant ongoing research [12]. Such experimentations and models in multimedia and human activity recognition—including hierarchical classification, deep learning, and transfer learning from already running dataset—approaches can be adopted [12].

The developmental perspectives from such IoT-driven research aim at designing such an architecture by full-time monitoring of older adult’s daily activities via smart home sensors, accompanied by reduction costs because of early diagnosis of diseases and accidents. By considering the vitality of the elderly health-care system, quality attributes that are met in the architecture are availability, performance, security, and interoperability. This architectural design of the system provides the reusability attribute and enhances the understanding attribute by reducing the complexity of the system design [48].

Acknowledging the reality that elderly persons face declining cognitive and physical capabilities as they advance in years, IoT sensor is an invaluable tool of the sensing system to watch over seniors as they go about their daily lives. The ideal operability of such a system has to work well and unobtrusively in an actual home environment, monitoring behavioral changes over a long period, thereby being used to detect abnormal statuses of other elderly residents via individualizing long-term monitoring programs [51].

The limitations of the current study rely on the quality of the synthetic dataset used for the purpose of this research. Specifically, the same models could exploit the potentiality of a real dataset and output a better result. At this stage this is a methodological limitation, which however is common in empirical sciences such as machine learning, which rely on empirical data. To overcome this limitation, we aim to generate real data from senior participants to test our models in future studies. In addition, to be able to compare equally all the examined models, we are going to test them with the real dataset in further research.

## 6. Conclusions and Future Research Orientations

This research investigated fall and lean over incidents of seniors in smart homes. We performed experiments with anonymous real and synthetic data from the Internet (YouTube videos) for training and testing the proposed temporal models. The most suitable model was the CM-II, including the variables of fall time and height, to classify an incident as fall or lean over. The results of the research are promising to infer and provide early warning of a fall incident, since 98% of all fall cases were correctly classified. Clinical doctors can exploit such knowledge with access to the temporal models to provide first aid services to seniors. 

In deeply understanding the algorithms that have the potential to improve healthcare systems based on IoT, a certain amount of such algorithms are already implemented. However, knowing the dependency on AI and deep learning, there is a high chance of minimum human errors, while with the use of standardized data sets over unstandardized data sets, the performance of training algorithms can be enhanced, thereby reducing complexity and computation time [4]. Moreover, various quality attributes scenarios can be drawn and discussed in order to extract quality attributes requirements. Therefore, a comprehensive evaluation of such IoT-driven architecture must be tested and verified. Then, risks’ detection, which causes undesirable results in quality attributes requirements, can enable future works’ integration of the older person’s healthcare system with smart city and municipality services [48]. Similar research proposals are oriented to focus groups and usability tests, showing that many users can be satisfied with the straightforward use of such an IoT model, reporting flexibility and reliability in interacting with the system. Accuracy in the movement of the pointer, make this model a reliable solution to simplify several daily tasks [52]. 

In conclusion, it is of the utmost importance to note that whenever young healthy subjects simulate falls without any impairment for safety reasons, researchers should be aware that some differences can be found with real falls in older people. Therefore, research experimentation and modeling cannot guarantee that fall prediction for older or impaired adults can be made with a model built directly using developed dataset. Hence, such dataset can be used for transfer learning experiments for prediction in elderly people or adults with impairments [12].

*In data collection, all activities were performed in the same order and trials were performed consecutively. Falls were self-initiated, and subjects fell onto a protective mattress that damped the impact of the simulation. This is a difference between real falls which generally occur towards hard materials and no intuitive reaction trying not to fall was recorded*.

[12]

*It is essential to notice that this dataset was thought for simple and non-overlapping activities, so down-sampling rates in IMUs (18 Hz) do not affect stationary fall predictions. This might be a limitation if the dataset would be used for real-life predictions during dynamic situations (e.g., concurrent falls-and-activities)*.

[12]

## Figures and Tables

**Table 1:** Classification Model I.

#	CM-I Model
1	**Input:**$N\;\left(\mu_{F},\sigma_{F}\right)$, $N\;\left(\mu_{B},\sigma_{B}\right)$
2	**Output:** Class
3	$read(t_{I})$//Incident with certain time
4	$r=\frac{\mu_{F}+\mu_{B}}{2}$
5	**While** (True) **Do**
6	**If** ($t_{I}<r$) **Then**
7	Class ← fall
8	**Else**
9	Class ← bend
10	**End If**
11	**End While**

**Table 2:** Classification Model II.

#	CM-II Model
1	**Input:**$t_{F}$, $t_{B}$
2	**Output:** Class
3	$read(t_{I})$//Incident vector with certain time measurements
4	j←0
5	fall←0
6	bent←0
7	**For** (j:0→n) **Do**//Repeat until h=0 i.e., floor is reached
8	$t_{fall}^{j}=\left|t_{I}^{j}-t_{F}^{j}\right|$
9	$t_{bent}^{j}=\left|t_{I}^{j}-t_{B}^{j}\right|$
10	**If** $\left(t_{fall}^{j}<t_{bend}^{j}\right)$ **Then**
11	fall←fall+1
12	**Else**
13	bend←bend+1
14	**End If**
15	**End For**
16	**If** (fall>bend) **Then**
17	Class ← fall
18	**Else**
19	Class ← bend
20	**End If**

**Table 3:** Experimental parameters.

Parameters	Values
Number of incident videos	86
Number of fall videos	41
Number of lean over videos	45
Evaluation method	10-fold cross validation
Video standard	NTSC
Frames per second (fps)	30
Height h interval (cm)	[0, 1.72]
Normal PDF of $t_{F}$ (seconds)	$N_{F}\;\left(1.3,\;0.39\right)$
Normal PDF of $t_{B}$ (seconds)	$N_{B}\;\left(2.1,\;0.48\right)$
Criterion r (seconds)	1.7
Metric a interval (net number)	[0, 1]
Metric $a^{\prime }$ interval (net number)	[0, 1]

**Table 4:** Model comparison.

Model	Accuracy
[10]	0.95
[15]	0.94
[17]	0.91
[18]	0.86
[22]	0.96
[24]	0.97
[25]	0.89
CM-II	0.98
